# Identification of penaeid shrimp from Chilika Lake through DNA barcoding

**DOI:** 10.1080/23802359.2018.1437794

**Published:** 2018-02-09

**Authors:** Shantanu Kundu, Shibananda Rath, Kaomud Tyagi, Rajasree Chakraborty, Vikas Kumar

**Affiliations:** Centre for DNA Taxonomy, Molecular Systematics Division, Zoological Survey of India, Kolkata, India

**Keywords:** Brackish water ecosystem, Crustaceans, Penaeidae, mtCOI, Gene pool

## Abstract

Chilika Lake is one of the prolific habitats of shrimps in India and offers tons of commercial trading every year. The genetic diversity of penaeid shrimp species in this oldest and largest brackish water lagoon is unknown so far. The DNA barcoding is emerging as an essential supportive tool for morphology-based species identification. In this study, we have generated DNA barcode data of morphologically identified six penaeid shrimps from Chilika Lake. Most of the generated sequences revealed 99–100% similarities with the conspecific database sequences (GenBank and BOLD). More than one distinct clade in NJ tree and high-genetic variability were resulted in *P. monodon* (6.5% to 8.8%), *L. vannamei* (3.2% to 5.8%) and *M. monoceros* (2.3% to 3.5%). The resulted genetic variation within the species depicted different population correlate with the different sampling locations. Thus, more extensive survey and generation of more DNA barcode data of penaeid shrimp from the diverse geographical area might resolve the uncertain genetic distance within the species.

## 1. Introduction

Chilika Lake is one of the largest brackish water lagoon in the east coast of India and second largest lagoon in the world. Geological data evidenced that, the coastal lagoon was part of Bay of Bengal during the later stages of the Pleistocene period. Due to several geological factors and climatic change, the lagoon was split from the Bay of Bengal and connected to the sea by an irregular channel. Further, the linking freshwater rivers and stream into the lake form a part freshwater character and that allows proliferation of an amazing number of species diversity. A major survey by Zoological Survey of India in 1985–88, revealed over 800 vertebrate fauna recorded from this oldest aquatic ecosystem, covering an area of over 1100 km^2^ (WWF 2008). Several fishermen communities sustain their livelihood by catching and trading the inhabiting aquatic bio-resources around this lake. However, in last two decades the extant fauna of Chilika Lake confront several natural and anthropogenic threats. A number of marine, brackish water and freshwater biota of this lake are now listed in the endangered, threatened and vulnerable categories (IUCN 2017). Thus, the monitoring of ecological changes and other related factors as well as intervention of molecular techniques for accurate species-level identification and their conservation measures are urgently required.

This brackish water ecosystem is also known as a prolific habitat of Crustacean biodiversity, with six species of penaeid shrimps recorded so far (Reddy 1995), viz., *Penaeus monodon, Penaeus semisulcatus, Fenneropenaeus indicus*, *Metapenaeus monoceros*, *Metapenaeus affinis,* and *Metapenaeus dobsoni*. The shrimps are ecologically and economically important species as they play a significant role in the ecosystem as well as highly traded as one of the lucrative seafood (Jayachandran 2001). Every year tons of penaeid shrimps are traded from this lake, nevertheless, due to several anthropogenic threats, the native population are frequently affected by diseases and loss the economic value (Rath and Dev Roy 2009). Furthermore, owing to the expanding distributions of shrimps in marine eco-system, invasive species often invade the indigenous species eco-system and possess competition in the same niche (Wakida-Kusunoki et al. 2011). The morphology-based species identification and estimates of the diversity of penaeid shrimps, is difficult because of their morphological variations in different life stages, phenotypic plasticity and sexual dimorphism (PrasannaKumar et al. 2012). Hence, the morphology-based assessment frequently misleads the species identification and thus increases the risks of seafood fraud (Nicolè et al. 2012; Maralit et al. 2013).

The partial fragment (∼650bp) of mitochondrial DNA (mtDNA), the cytochrome C oxidase subunit I (COI) gene has been standardized to identify the penaeid shrimps (Rajkumar et al. 2015; Jose et al. 2016; Saad and El-Sadek 2017). Different molecular-based approaches also have been tested to identify the commercialized products of shrimps to confirm their origin (Besbes et al. 2015). So far, several studies were aimed to determine the diversity of shrimps from southern part of India through DNA barcoding approaches (Mamatha et al. 2016; Subbaiya et al. 2017). However, the DNA-based investigation of penaeid shrimps has never been attempted from Chilika Lake. Hence, the research work first aimed to determine the efficacy of mitochondrial cytochrome oxidase subunit I gene (mtCOI) gene to identify the taxonomically identified penaeid shrimps from Chilika Lake and also evaluate the genetic variability. This baseline integrative approach would substantiate the further taxonomic research on penaeid shrimps from India and other regions. The generated barcode data would enrich the global database, help to estimate the population structure of morphologically static species and also detect the commercial seafood fraud.

## 2. Materials and methods

### 2.1. Sampling and morphological identification

The penaeid shrimps were collected from Nalabana island of Chilika Lake (19.69 N 85.29 E) in eastern coast of Odisha state. The collected specimens were identified by available keys (Reddy 1995; Isabel 1997; Dholakia 2010). The specimens were preserved in 70% alcohol and deposited in the Crustacea Section of Zoological Survey of India, Kolkata.

### 2.2. Genomic DNA isolation, PCR and sequencing

The total genomic DNA was extracted from the muscle tissue in 500 µl ATL buffer containing 50mM Tris-HCl (pH 8.0), 25mM EDTA (pH 8.0), and 150 mM NaCl by Proteinase K (200 µg/ml) with standardized Phenol-chloroform extraction method (Sambrook and Russell 2001). The extracted DNA is checked by 1.5% Agarose gels electrophoresis using standard protocol. For amplification of mtCOI segment, the published Primer pair was used (Folmer et al. 1994). The 25µl PCR reaction mixture contains 10 pmol of each primer, 10–20 ng of DNA template, 1× PCR buffer, 1.0–1.5 mM of MgCl2, 0.25 mM of each dNTPs, and 0.25 U of high-fidelity TaqDNA polymerase. The thermal profile for PCR was set as initial denaturation at 94 °C for 2 min, followed by 30 cycles at 94 °C for 45 s, 50 °C for 45 s and 72 °C for 1 min, and subsequent storage at 4 °C and amplification was performed using a Veriti® Thermal Cycler. The PCR product was purified using QIAquickR Gel extraction kit and cycle sequencing products were cleaned by using standard BigDye X Terminator Purification Kit. The bidirectional sequencing was generated by the 48 capillary array Applied Biosystems 3730 DNA Analyzer in the in-house sequencing facilities in the Zoological Survey of India, Kolkata. The generated sequences were checked by Sequence Analysis software (ABI) and assured by online BLAST search program and ORF finder. Finally the generated sequences were submitted in global database (GenBank) to acquire the specific accession number.

### 2.3. Dataset preparation and sequence analysis

We screened the GenBank database to acquire the publicly available COI sequences of penaeid shrimps (family Penaeidae). The bi-directional chromatograms of the generated sequences were checked and the noisy parts were trimmed at both the ends to avoid the noisy part. The nucleotide BLAST (BLASTn) program was used to evaluate the sequences. The screened fragments were aligned using ClustalX software (Thompson et al. 1997) and finally, each of the sequences was compared in NCBI through BLASTn and ORF finder to examine the complete alignment and stop codons (http://www.ncbi.nlm.nih.gov/gorf/gorf.html). Primarily, the generated sequences were identified at the online identification system, in GenBank with ‘Highly similar sequences (megablast)’ and BOLD databases with ‘All Barcode Records on BOLD’. The total dataset consists 16 generated sequences of six morphologically identified species, viz., *P. monodon*, *F. indicus*, *Litopenaeus vannamei*, *M. dobsoni*, *M. monoceros*, *Metapenaeus ensis* and 44 representative sequences (including one out-group) of same and related taxa from the database. The mtCOI sequences were analyzed through Neighbour-Joining (NJ) tree and Kimura 2 parameter (K2P) by using MEGA6 to infer the genetic distance and monophyletic clustering of the studied taxa (Tamura et al. 2013).

## 3. Results and discussion

**Figure 1.**
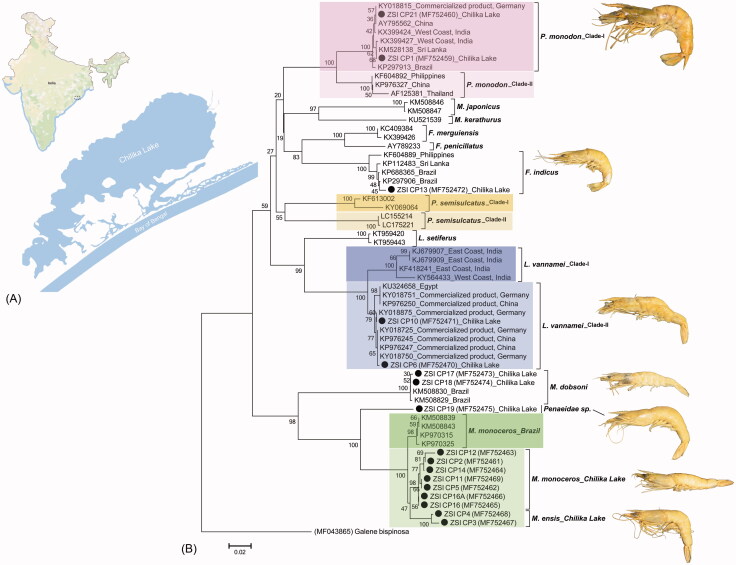
(A) Collection locality of the studied penaeid shrimps species from Chilika Lake of eastern India. (B) Neighbour-joining (NJ) tree of the studied penaeid shrimps with bootstrap support. The crab species, *Galene bispinosa* used as an out-group in the phylogeny. The black dots represent the generated sequences in this study. The collection localities of the generated and database sequences were also mentioned in the tree after accession numbers and species name. Colour bars (light and deep) show the ambiguous clade of some penaeid shrimp species in the present study correlate to the high-genetic variability.

The generated sequences of the studied penaeid shrimp species from Chilika Lake were annotated (616bp) and submitted into the global database (GenBank) to acquire the unique accession numbers. Most of the generated sequences show 99–100% similarities with the conspecific database sequences in both GenBank and BOLD. However, the studied sample (ZSI_CP19) was unable to identify due to lack of morphological characters and classify up to family level, as Penaeidae species based on similarity search results in both GenBank and BOLD database. The estimated NJ tree depicted cohesive clustering of the dataset sequences with high bootstrap support (Figure 1). However, the ZSI_CP19 sample shows distinct clade within *Metapenaeus* genus in NJ tree and shows 20.4–20.6% genetic divergence with *M. dobsoni* (Table 1). Further, the ZSI_CP19 also shows 9–11.7% genetic divergence with *M. monoceros* and *M. ensis*, that evidenced sister species of *M. monoceros* and *M. ensis*.

**Table 1.** Average evolutionary K2P genetic divergence over sequence pairs between and within groups of the studied penaeid shrimps species group as resulted in the NJ phylogeny.

	Between groups																Within groups
*P. monodon*_Clade-I																	0.3
*P. monodon*_Clade-II	7.5																0.8
*M. japonicus*	19.6	19.6															0.3
*M. kerathurus*	16.9	17.7	15.7														n/c
*F. merguiensis*	15.7	18.0	18.6	18.5													0.3
*F. penicillatus*	16.1	16.7	18.1	18.5	6.9												n/c
*F. indicus*	17.1	16.2	18.0	21.1	13.6	14.8											1.2
*P. semisulcatus*_Clade-I	16.0	17.5	20.3	19.0	16.3	16.4	18.1										1.9
*P. semisulcatus*_Clade-II	16.7	18.2	20.2	20.2	18.0	17.3	17.7	15.9									0.1
*L. setiferus*	17.9	17.2	19.2	21.0	18.3	17.2	18.2	19.0	20.2								0.3
*L. vannamei*_Clade-I	21.1	21.2	22.7	22.2	19.8	20.9	20.4	20.6	19.5	16.0							1.5
*L. vannamei*_Clade-II	18.0	18.1	20.9	20.8	18.8	20.1	19.0	17.4	18.8	12.4	4.5						0.4
*M. dobsoni*	22.4	23.7	23.8	25.7	24.2	24.5	25.0	24.3	27.6	24.4	23.6	23.4					0.1
Penaeidae_sp.	23.4	23.2	22.5	24.1	23.3	23.5	23.3	22.6	25.1	22.4	25.5	23.5	20.5				n/c
*M. monoceros*_Brazil	23.2	22.4	24.1	23.5	23.2	23.4	22.6	23.2	23.1	23.5	27.6	25.7	19.4	9.2			0.2
*M. monoceros*_Chilika Lake	23.7	23.5	24.5	23.8	24.1	24.7	22.9	24.5	23.9	23.5	27.6	25.3	19.9	11.6	2.7		0.6
*M. ensis*_Chilika Lake	24.1	23.3	25.4	25.0	24.5	25.4	22.6	25.3	24.8	24.9	29.8	27.4	21.2	11.2	3.2	3.5	0.8

n/c: not able to calculate due to single sequence. High K2P genetic divergences within the species are highlighted by grey colour.

The *F. indicus* and *M. dobsoni* showed distinct clade with the database sequences with 0.2% to 2.5% and 0% to 0.3% K2P genetic divergence, respectively (Table 1), however, the *P. monodon*, *L. vannamei*, *M. monoceros* and *M. ensis* showed ambiguous clustering with the publically available database sequences. The NJ tree depicted the *Marsupenaeus japonicus* and *Melicertus kerathurus* as sister species in the dataset. However, *Fenneropenaeus merguiensis* and *Fenneropenaeus penicillatus* are close to the *F*. *indicus*, as belonging to the same genus. The four database sequences of *Penaeus semisulcatus*, resulted two different clades in the NJ tree and not discussed further. The sequences of *Litopenaeus setiferus* resulted sister clade with *L. vannamei* in the present NJ phylogeny. The Kimura-2-parameter (K2P) genetic divergence was further calculated to resolve the ambiguous clade as resulted for the penaeid shrimps collected from the studied locality. Each species with ambiguous clade are discussed below with their collection localities and assumed their variation in genetic level.

It is evident that the mtCOI is a successful partial gene segment to construct the phylogeny of crustacean species (Saad and El-Sadek 2017). In this study, *Penaeus monodon* shows two distinct clade in NJ tree, one clade (*P. monodon*_Clade-I) consisting of eight sequences, generated from Germany (commercialized products), Brazil, Sri Lanka, China, West Coast of India, Chilika Lake and the other clade (*P. monodon*_Clade-II) consisting of three sequences, generated from Philippines, China and Thailand (Figure 1). The K2P genetic divergence within the *P. monodon*_Clade-I is ranging from 0% to 0.8% and *P. monodon*_Clade-II is ranging from 0% to 1.3%. However, the *P. monodon*_Clade-I and *P. monodon*_Clade-II shows 6.5% to 8.8% genetic divergence (Table 1). Thus, the resulted high-genetic divergence between the two clades in the dataset might depict two different population of *P. monodon* within or outside of the collection localities.

The white leg shrimp, *L. vannamei* has been popularly known as one of the successful species in Indian aquaculture. This pathogen-free species has shown phenomenal farming growth and reported production of 2.80 lakh tones during 2012–2013 (CIBA Annual Report, 2013–2014). Although, *L. vannamei* is native to the tropical East Pacific from the Gulf of California, Mexico to northern Peru; but presently regarded as one of the most widely cultured shrimp in the world (Holthuis 1980; Liao & Chien 2011). In the present dataset, the *L. vannamei* shows two clades in NJ tree, one clade (*L. vannamei*_Clade-I) consisting of four sequences; generated from both east and west coast of India. However, the remaining 10 sequences were generated from Egypt, Germany (commercialized products), China (commercialized products) and Chilika Lake. The *L. vannamei*_Clade-I and *L. vannamei*_Clade-II also shows high genetic divergence (3.2% to 5.8%) and might depicted two different population of *L. vannamei*.

Further, the *M. monoceros* and *M. ensis* often possess difficulties in species-level identification due to overlapping morphological characters. The present study identified three specimens (ZSI_CP11, ZSI_CP3 and ZSI_CP4) as *M. ensis*, however, the molecular data resulted, ZSI_CP11 as *M. monoceros* in NJ tree.The two specimens, ZSI_CP3 and ZSI_CP4 shows distinct clade with high bootstrap support and sufficient genetic divergence (2.8% to 4.7%) with *M. monoceros*. Although, one specimen (ZSI_CP11) of *M. ensis* shows an equivocal result, the morphologically identified two *M. ensis* specimens (ZSI_CP3 and ZSI_CP4) confirms the genetic distinctiveness of *M. monoceros* and *M. ensis* by partialmtCOIregion. Moreover, the generated sequences of *M. monoceros*, collected from Chilika Lake resulted distinct clade in NJ tree and high genetic divergence (2.3% to 3.5%) with the database sequences, generated from Brazil (Figure 1). Hence, the genetic distinctiveness within *M. monoceros*, assumed to be two different population, one from the South Atlantic ocean and other from the Indian ocean. The study revealed that the *M. monoceros* population of Chilika Lake may represent restricted gene pools, which need to be re-evaluated thoroughly.

The genetic variability or evenness of shrimp species have been tested by mtCOI gene and the observed variations were correlated to the geographical isolation (Vergamini et al. 2011; Rossi and Mantelatto 2013). Thus, the resulted ambiguous genetic variability within *P. monodon*, *L. vannamei* and *M. monoceros* in this present study, further compel more extensive sampling and generation of more DNA barcode data of studied species from different geographical localities to resolve the genetic dissimilarity. Besides, the invasion of non-native gene pool may possess threat to the indigenous penaeid shrimp population in Chilika Lake. Thus, to monitor and protect the native species in this eco-system and prevent the ingress of exotic taxa, both morphological and DNA-based species assessment may be adopted eventually.
